# Pharmacological Inhibition of Host Heme Oxygenase-1 Suppresses *Mycobacterium tuberculosis* Infection *In Vivo* by a Mechanism Dependent on T Lymphocytes

**DOI:** 10.1128/mBio.01675-16

**Published:** 2016-10-25

**Authors:** Diego L. Costa, Sivaranjani Namasivayam, Eduardo P. Amaral, Kriti Arora, Alex Chao, Lara R. Mittereder, Mamoudou Maiga, Helena I. Boshoff, Clifton E. Barry, Celia W. Goulding, Bruno B. Andrade, Alan Sher

**Affiliations:** a Immunobiology Section, Laboratory of Parasitic Diseases, NIAID, NIH, Bethesda, Maryland, USA; b Tuberculosis Research Section, Laboratory of Clinical Infectious Diseases, NIAID, NIH, Bethesda, Maryland, USA; c Department of Molecular Biology and Biochemistry, University of California, Irvine, Irvine, California, USA; d Department of Pharmaceutical Sciences, University of California, Irvine, Irvine, California, USA; e Unidade de Medicina Investigativa, Laboratório Integrado de Microbiologia e Imunorregulação, Instituto Pesquisas Gonçalo Moniz, Fiocruz, Salvador, Bahia, Brazil; f Multinational Organization Network Sponsoring Translational and Epidemiological Research, Instituto Brasileiro para a Investigação da Tuberculose, Fundação José Silveira, Salvador, Bahia, Brazil

## Abstract

Heme oxygenase-1 (HO-1) is a stress response antioxidant enzyme which catalyzes the degradation of heme released during inflammation. HO-1 expression is upregulated in both experimental and human *Mycobacterium tuberculosis* infection, and in patients it is a biomarker of active disease. Whether the enzyme plays a protective versus pathogenic role in tuberculosis has been the subject of debate. To address this controversy, we administered tin protoporphyrin IX (SnPPIX), a well-characterized HO-1 enzymatic inhibitor, to mice during acute *M. tuberculosis* infection*.* These SnPPIX-treated animals displayed a substantial reduction in pulmonary bacterial loads comparable to that achieved following conventional antibiotic therapy. Moreover, when administered adjunctively with antimycobacterial drugs, the HO-1 inhibitor markedly enhanced and accelerated pathogen clearance. Interestingly, both the pulmonary induction of HO-1 expression and the efficacy of SnPPIX treatment in reducing bacterial burden were dependent on the presence of host T lymphocytes. Although *M. tuberculosis* expresses its own heme-degrading enzyme, SnPPIX failed to inhibit its enzymatic activity or significantly restrict bacterial growth in liquid culture. Together, the above findings reveal mammalian HO-1 as a potential target for host-directed monotherapy and adjunctive therapy of tuberculosis and identify the immune response as a critical regulator of this function.

## IMPORTANCE

There is no reliable vaccine against tuberculosis (TB), and conventional antibiotic therapy is administered over at least 6 months. This prolonged treatment period can lead to noncompliance resulting in relapsed infection as well as the emergence of multidrug resistance. Thus, there is an urgent need for improved therapeutic regimens that can more rapidly and efficiently control *M. tuberculosis* in infected patients. Here, we describe a potential strategy for treating TB based on pharmacological inhibition of the host heme-degrading enzyme HO-1. This approach results in significantly reduced bacterial burdens in mice, and when administered in conjunction with conventional antibiotic therapy, leads to faster, more effective pathogen clearance without detectable direct effects on the mycobacteria themselves. Interestingly, the effects of HO-1 inhibition on *M. tuberculosis* infection *in vivo* are dependent on the presence of an intact host immune system. These observations establish mammalian HO-1 as a potential target for host-directed therapy of TB.

## OBSERVATION

*Mycobacterium tuberculosis* is now regarded as the world’s leading cause of death due to a single infectious agent. While effective chemotherapy exists for the treatment of tuberculosis (TB), the standard antibiotic regimens must be administered for a minimum of 6 months, and noncompliance can lead to disease reactivation together with the emergence of multidrug-resistant bacterial strains (1). In the absence of a reliable vaccine, the development of new therapeutic approaches that can more effectively and rapidly control *M. tuberculosis* are greatly needed to reduce the current global disease burden.

Host-directed therapy (HDT) has a unique advantage for achieving this goal in that, by targeting host factors that play crucial functions during the infectious process rather than targeting the pathogen itself, HDT should not promote the development of drug-resistant bacteria. In the case of TB, a number of different HDT approaches have been proposed or are in clinical trials, and are being tested for their ability to accelerate conventional chemotherapy or treat multidrug-resistant infections (2).

In this study, we identify heme oxygenase-1 (HO-1) as a potential target for HDT of TB. This antioxidant enzyme, which catalyzes heme degradation into biliverdin, iron, and carbon monoxide (3), is induced during both experimental and clinical *M. tuberculosis* infection, and its production is reduced following successful antibiotic treatment (4–7). Previous studies have shown that mice genetically deficient for HO-1 are more susceptible to *M. tuberculosis* infection (8). However, the interpretation of the latter finding is complicated by the presence of prominent hematopoietic abnormalities in these animals (9, 10). Moreover, in sera from TB patients, HO-1 levels are positively rather than negatively correlated with disease severity (6). These observations led us to test the function of HO-1 on experimental TB by administering a widely utilized pharmacological inhibitor of the enzyme, tin protoporphyrin IX (SnPPIX), to *M. tuberculosis*-infected C57BL/6 mice.

### Methods. (i) Mice and *M. tuberculosis* infections.

C57BL6 and TCR-α^−/−^ mice were purchased from Taconic Farms (Germantown, NY, USA). All animals were housed at biosafety level 2 (BSL-2) and BSL-3 animal facilities at the National Institute of Allergy and Infectious Diseases (NIAID), National Institutes of Health (NIH), and all experiments utilized protocols approved by the NIAID Animal Care and Use Committee. Mice were infected with approximately 100 CFU of the H37Rv strain of *M. tuberculosis* by using an aerosol chamber (Glas Col, Terre Haute, IN, USA). Determination of bacterial loads was performed by culturing serial dilutions of tissue homogenates in 7H11 medium (Sigma-Aldrich, St. Louis, MO, USA) supplemented with oleic acid-albumin-dextrose-catalase (BD Biosciences, San Diego, CA, USA).

Additional information on the materials and methods used in our study can be found in Text S1 in the supplemental material.

### (ii) Antibiotic and SnPPIX treatments.

The antibiotics rifampin (R; 10 mg/kg of body weight/mouse), isoniazid (H; 25 mg/kg/mouse), and pyrazinamide (Z; 150 mg/kg/mouse) (all from Sigma-Aldrich) were used. *M. tuberculosis*-infected mice were treated with a cocktail of RHZ during the first 60 days of treatment and with RH thereafter. The drugs were administered by gavage 5 days per week, and fresh stock solutions were prepared weekly. The heme oxygenase-1 inhibitor SnPPIX (Frontier Scientific, Logan, UT, USA) was administered by daily intraperitoneal injection (5 mg/kg/mouse). The drug was dissolved in 0.1 M NaOH aqueous solution and then diluted in 10× phosphate-buffered saline (PBS), with further pH adjustment to 7.0 to 7.4. Aliquots were frozen at −80°C and thawed immediately prior to inoculation.

### (iii) Quantification of *Hmox1* mRNA expression by real-time PCR.

mRNA was extracted from lungs of *M. tuberculosis*-infected and naive mice by using Trizol reagent (Invitrogen/Thermo Fisher Scientific, Waltham, MA, USA), and RNeasy minikits (Qiagen, Hilden, Germany). cDNA was reverse transcribed using 1 µg of RNA, SuperScript II reverse transcriptase, and random primers (all from Invitrogen/Thermo Fisher Scientific). SYBR green and 7900HT fast real-time PCR systems (Applied Biosystems/Thermo Fisher Scientific) were employed for real-time PCRs. The relative expression of HO-1 in *M. tuberculosis*-infected mouse lungs was calculated using the ΔΔ*C_T_* (cycle threshhold) method, normalizing mRNA expression in each sample to that of β-actin, and further comparing them in relation to expression in uninfected naïve mouse lungs. The primer sequences used are provided in Table S2 in the supplemental material.

### (iv) HO-1 measurement in lung homogenates by using Western blotting.

Briefly, *M. tuberculosis*-infected and naïve mouse lungs were perfused with PBS and homogenized in PBS containing Complete Ultra protease inhibitor cocktail (Roche, Basel, Switzerland) and 2 mM phenylmethylsulfonyl fluoride (Sigma Aldrich). The protein concentrations from all samples were normalized, and then reducing buffer (Pierce/Thermo Fisher Scientific) was added to samples prior to incubation for 5 min at 95°C for protein denaturation. Samples were separated in Mini-Protean TGX gels (Bio-Rad, Hercules, CA, USA) and transferred to polyvinylidene difluoride membranes prior to staining with anti-mouse HO-1 (SPA-895; Enzo Life Sciences, Farmingdale, NY, USA) or anti-mouse glyceraldehyde-3-phosphate dehydrogenase (GAPDH; ab9485; Abcam, Cambridge, MA, USA) and anti-rabbit IgG conjugated to horseradish peroxidase (catalog number 7074; Cell Signaling Technology, Danvers, MA, USA).

### (v) Statistical analyses.

Differences between groups were statistically evaluated by using an unpaired Student *t* test (based on a parametric distribution of the data) within Prism software (GraphPad, San Diego, CA, USA), and differences were considered significant when *P* was ≤0.05.

### Findings.

**FIG 1:**
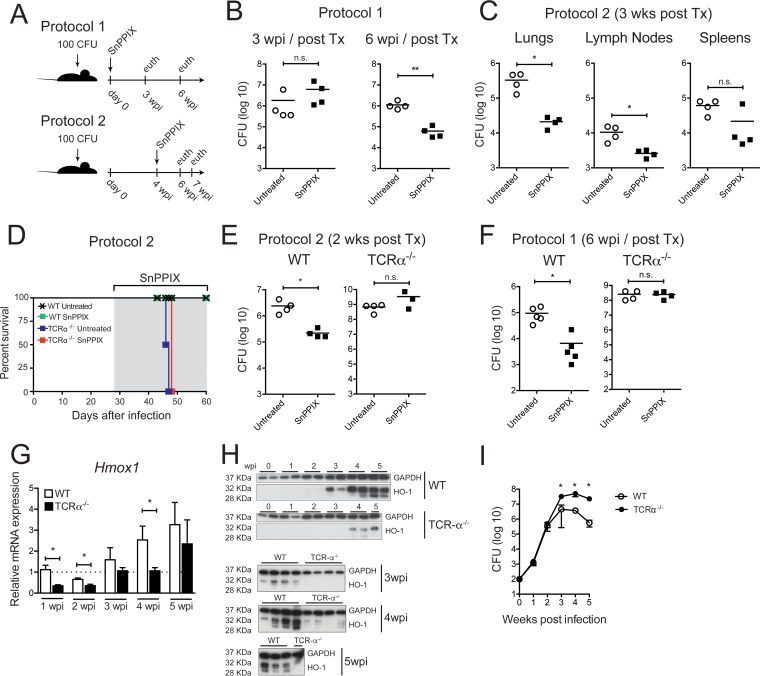
Treatment with SnPPIX results in decreased pulmonary bacterial loads in *M. tuberculosis*-infected mice with an intact T cell compartment. (A) Diagram of the two experimental protocols employed for infection and SnPPIX treatment. (B) CFU in lungs of *M. tuberculosis*-infected C57BL/6 (WT) mice with or without SnPPIX treatment (protocol 1) at 3 and 4 wpi. (C) CFU in lungs, mediastinal lymph nodes, and spleens of *M. tuberculosis*-infected WT mice either treated or not with SnPPIX (protocol 2), assayed at 3 weeks post-treatment initiation. (D) Survival curve of *M. tuberculosis*-infected WT and TCR-α^−/−^ mice with or without SnPPIX treatment (protocol 2). (E) CFU in lungs of *M. tuberculosis*-infected WT and TCR-α^−/−^ mice with or without SnPPIX treatment (protocol 2) assayed at 2 weeks post-treatment initiation. (F) CFU in lungs of *M. tuberculosis*-infected WT and TCR-α^−/−^ mice treated or not with SnPPIX (protocol 1), measured at 6 wpi. (G) HO-1 mRNA expression, measured by real-time PCR in lungs of *M. tuberculosis*-infected WT and TCR-α^−/−^ mice at 1, 2, 3, 4, and 5 wpi. (H) Quantification of HO-1 and GAPDH protein expression by Western blotting assays of lungs of uninfected (0 wpi) or *M. tuberculosis*-infected WT and TCR-α^−/−^ mice at 1, 2, 3, 4, and 5 wpi (2 top images, WT and TCR-α^−/−^ samples, respectively, in separate gels) and at 3, 4, and 5 wpi (3 bottom images, WT and TCR-α^−/−^ samples in the same gel). (I) CFU in lungs of *M. tuberculosis*-infected WT and TCR-α^−/−^ mice assayed at day 1 (0 wpi) and 1, 2, 3, 4, and 5 wpi. Graphs show individual results or means ± standard deviations of results. *, *P* ≤ 0.05; **, *P* ≤ 0.01. n.s., nonsignificant. Each experimental group consisted of 4 to 5 mice. Each panel shows the results of a representative experiment of 2 to 4 performed.

When given to animals by daily intraperitoneal injection beginning on the same day as aerosol *M. tuberculosis* infection (Fig. 1A, protocol 1), SnPPIX induced a highly significant reduction in pulmonary bacterial load that was evident at 6 weeks but not 3 weeks postinfection (wpi) (Fig. 1B). A similar reduction was achieved when SnPPIX treatment was initiated at 4 wpi (Fig. 1A, protocol 2, and Fig. 1C), and bacterial burdens were measured 3 weeks later. Interestingly, the effects of SnPPIX administration were more prominent in the lungs than in mediastinal lymph nodes or spleens (Fig. 1C).

The pathogen-specific T cell response in lungs of *M. tuberculosis*-infected mice is known to be delayed until approximately 3 wpi (11). This suggested to us that the delay in efficacy of SnPPIX on murine *M. tuberculosis* infection might be due to a requirement for T lymphocyte cooperation in the activity of the HO-1 inhibitor. To test this hypothesis, we administered SnPPIX to *M. tuberculosis*-infected C57BL/6 (wild type [WT]) and T cell receptor α-deficient (TCR-α^−/−^) mice (which lack conventional TCR-αβ^+^ CD4 and CD8 T cells) beginning at 4 weeks postbacterial exposure (Fig. 1A, protocol 2). SnPPIX treatment failed to protect the infected TCR-α^−/−^ mice, which presented similar mortality kinetics as untreated TCR-α^−/−^ mice (Fig. 1D). In parallel experiments in which animals were euthanized at 2 weeks after treatment initiation, SnPPIX-treated infected TCR-α^−/−^ mice displayed bacterial loads indistinguishable from those in untreated TCR-α^−/−^ animals, while WT animals treated with the compound showed a highly significant reduction in mycobacterial burden compared to untreated control mice (Fig. 1E). To control for the higher bacterial burden expected in TCR-α^−/−^ mice at 4 wpi, we performed a separate set of experiments in which we initiated SnPPIX administration on the same day as infection (Fig. 1A, protocol 1) and then evaluated pulmonary bacterial load after 6 weeks of treatment. As expected, WT mice receiving SnPPIX displayed a significant reduction in pulmonary bacterial loads, while among the surviving TCR-α^−/−^ mice no difference in bacterial burden was observed (Fig. 1F). These results indicated that the efficacy of SnPPIX on *M. tuberculosis* infection is dependent on host T cells and indirectly argue against a possible direct effect of the inhibitor on the bacteria themselves.

Additional experiments were then performed to formally rule out the possibility that SnPPIX is directly toxic for *M. tuberculosis*, a situation that might occur through its targeting of the bacterium’s own heme-degrading enzyme, MhuD, used by the pathogen for iron acquisition (12). To test this hypothesis, we first cultured bacteria in either iron-containing or iron-free GAST (glycerol-alanine-salts-Tween 80) liquid medium in the presence of increasing concentrations of SnPPIX over a 28-day period. No inhibition of bacterial growth was observed even at 125 µM SnPPIX in complete medium, while toxicity was observed at 125 µM in iron-free medium. This slight attenuation of growth was rescued in the presence of 10 µM hemin, arguing that the attenuation was unrelated to the inhibition of MhuD activity by SnPPIX (see Table S1 in the supplemental material).

When exposed to adverse conditions, such as low pH and oxygen concentrations, as well as to reactive oxygen or nitrogen species, *M. tuberculosis* undergoes changes in gene expression and metabolism that promote its survival in the harsh phagosomal environment of activated macrophages (13). In order to test whether such conditions might promote bacterial sensitivity to SnPPIX, we cultured *M. tuberculosis* in low-pH 7H9 medium in the presence of 100 µM sodium nitrite to simulate both acid and nitrosative stress from the intramacrophage compartment. Even at SnPPIX concentrations as high as 125 µM, no inhibition of bacterial growth was observed over a 21-day period in either the presence or absence of nitrite (see Table S1 in the supplemental material).

We next tested whether SnPPIX could be directly degraded by *M. tuberculosis* MhuD or inhibit its heme-cleaving activity. While MhuD-heme complexes underwent rapid degradation, MhuD-SnPPIX complexes remained stable for at least 24 h (see Fig. S1A in the supplemental material), demonstrating that SnPPIX is not cleaved by the bacterial enzyme. Moreover, when MhuD-heme complexes were incubated in the presence of SnPPIX at concentrations as high as 2 µM, no inhibition of heme degradation was observed (see Fig. S1B). In sharp contrast, the heme-degrading activity of mammalian HO-1 was completely blocked under the same conditions (see Fig. S1C). Together, these findings argue that the *in vivo* effects of SnPPIX on *M. tuberculosis* infection are unlikely to be due to a direct effect of the compound on the bacterium itself.

To further explore the requirement for T cells in the activity of SnPPIX on *M. tuberculosis* infection *in vivo*, we next asked whether induction of the host HO-1 is altered in T cell-deficient mice. In lungs of WT mice, increases in HO-1 gene expression were not detected until 3 wpi, and the protein, as measured by Western blotting, was first evident at the same time point (Fig. 1G and H). However, in lungs of TCR-α^−/−^ mice, HO-1 gene and protein expression were delayed until 4 to 5 wpi and were reduced relative to that observed in WT lungs (Fig. 1G and H), despite the increased bacterial loads present in the TCR-α^−/−^ animals (Fig. 1I). In direct contrast, expression of bacterial MhuD mRNA, if anything, was increased in the lungs of infected TCR-α^−/−^ versus WT mice (see Fig. S1D in the supplemental material), reinforcing the finding that *M. tuberculosis* MhuD is unaffected by SnPPIX and plays no role in the phenomena observed.

Together, the above results suggest that *M. tuberculosis* infection is refractory to SnPPIX treatment in T cell-deficient mice because of reduced pulmonary expression of host HO-1. The latter could result from either impaired recruitment of enzyme-expressing cells to the lungs or defective induction of enzyme synthesis because of the absence of a T cell response. In this regard, macrophages and monocytes rather than T cells have been shown to be the major source of HO-1 in infected lungs of WT mice as well as human lungs (14). While purified bone marrow-derived macrophage cultures can produce HO-1 in response to *M. tuberculosis* infection in the absence of T cells (7), it is possible that the infected tissue macrophage subpopulations in the lungs of *M. tuberculosis*-exposed mice require additional T cell activation signals to achieve optimal enzyme expression *in vivo*. Future work is needed to resolve the nature of the T cell requirement in SnPPIX function in order to better inform the use of this strategy as an HDT in TB patients.

**FIG 2:**
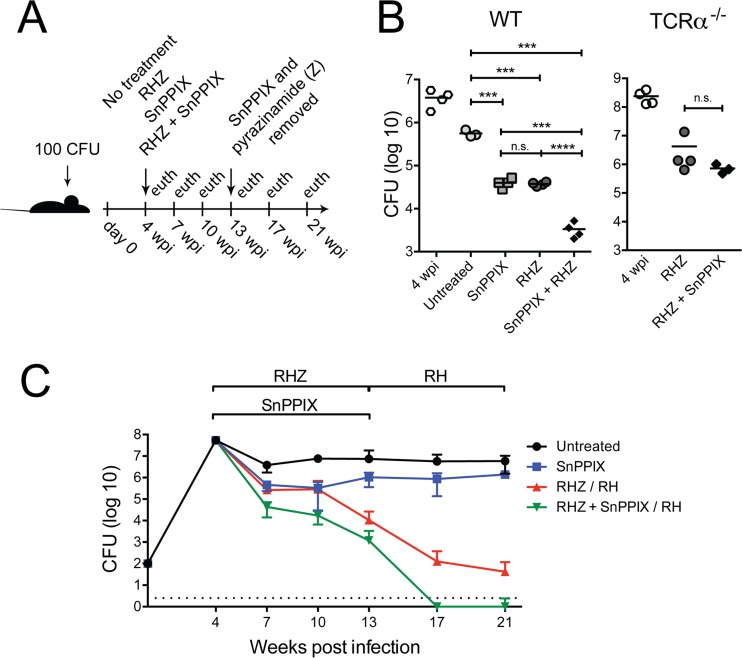
Administration of SnPPIX in conjunction with conventional antibiotic treatment accelerates pulmonary bacterial clearance in *M. tuberculosis*-infected mice. (A) Diagram of the experimental protocol employed. (B) Quantification of CFU in lungs of *M. tuberculosis*-infected C57BL/6 (WT) mice at day 0 of treatment (4 wpi) and 3 weeks post-treatment initiation, in which animals were left untreated or were administered RHZ, SnPPIX, or RHZ plus SnPPIX, or in lungs of TCR-α^−/−^ mice at day 0 of treatment (4 wpi) and 3 weeks post-treatment with RHZ or RHZ plus SnPPIX. (C) Quantification of CFU in lungs of *M. tuberculosis*-infected WT mice at the multiple time points described for panel A. The dotted line represents the limit of detection for the assay. Graphs show individual results or means ± standard deviations of the results. *, *P* ≤ 0.05; ***, *P* ≤ 0.001, n.s., nonsignificant. Each experimental group consisted of 4 to 5 mice. The experiment shown in panel B is representative of 2 to 3 performed, while the time course results shown in panel C are from a single experiment.

We next evaluated whether adjunctive administration of SnPPIX could enhance the efficacy of conventional anti-TB drug therapy. To do so, we treated infected mice at 4 weeks postinfection with either rifampin (R), isoniazid (H), and pyrazinamide (Z) alone (RHZ), SnPPIX alone, or a combination of SnPPIX plus RHZ, and we measured bacterial burdens after 3 weeks or later (Fig. 2A). The RHZ and SnPPIX treatments each resulted in an approximate 1-log reduction in pulmonary bacterial loads below those in untreated infected animals, and when combined these treatments resulted in an additive 2-log reduction in mycobacterial burden (Fig. 2B, left panel). As expected from our earlier experiments, the adjunctive treatment with SnPPIX failed to enhance RHZ efficacy in infected T cell-deficient mice (Fig. 2B, right panel). We then performed a time course experiment to evaluate the long-term consequence of combined SnPPIX-RHZ therapy. Interestingly, the major additive effects of SnPPIX on RHZ treatment were observed during the first 3 weeks of drug administration. Regardless, the combined therapy resulted in undetectable bacterial loads at 17 weeks from initiation of treatment, while mycobacteria were still detected as late as 21 weeks in mice treated with RHZ alone (Fig. 2C). Although effective when administered at the same time as antibiotic treatment, SnPPIX supplementation failed to enhance RHZ efficacy when initiated 6 weeks after the start of drug therapy (see Fig. S2A in the supplemental material). This outcome may have been due to a decline in host HO-1 expression following RHZ administration (see Fig. S2B), which was temporally correlated with both the reduction in bacterial burden and in the magnitude of the accompanying CD4 (see Fig. S2C) and CD8 (see Fig. S2D) T cell gamma interferon response at 3 weeks after initiation of RHZ therapy.

### Conclusions.

HO-1 is produced by host cells in response to oxidative stress and catalyzes heme degradation to iron, carbon monoxide (CO), and biliverdin. Bilirubin has potent antioxidant properties, while CO has cytoprotective effects (3). In addition, HO-1, by degrading heme, can suppress the inflammatory tumor necrosis factor-inducing and interleukin-1β (IL-1β)-inducing properties of that molecule (15) and has been shown to promote IL-10-mediated suppression (16). These anti-inflammatory effects can have paradoxical outcomes during the response to infection, as they can be host protective in some settings, such as experimental malaria (17) and Chagas’ disease (18), while detrimental in others, such as experimental melioidosis (19) and macrophage infection with *Leishmania chagasi* (20) or *Salmonella enterica* serovar Typhimurium (21). Such pathogen-promoting effects of HO-1 have been attributed to its suppression of the production of antimicrobial proinflammatory cytokines and metabolites by infected cells.

HO-1 production during *M. tuberculosis* infection was initially argued to be beneficial to the host, since CO released during heme degradation was shown to be toxic to the bacilli and to induce the expression of stress response genes (4, 5, 22). Consistent with these observations, mice with a genetically engineered deletion in the *Hmox1* gene displayed increased susceptibility to *M. tuberculosis* infection (8), although these animals are known to possess major secondary defects that affect the hematopoietic compartment, and they display spontaneous mortality (9, 10).

The results presented here, in which pharmacological blockade of HO-1 resulted in marked reduction in bacterial load, argue instead for a host-detrimental role for the enzyme in *M. tuberculosis* infection. This conclusion is supported by a recent study demonstrating that pharmacological inhibition of HO-1 in *M. tuberculosis*-infected human macrophages resulted in a reduction in intracellular bacterial loads as well as decreased proinflammatory cytokine production (14). Although we at present cannot rule out possible off-target effects of SnPPIX, our data indicate that direct toxicity of the compound for *M. tuberculosis* itself is highly unlikely. The mechanism(s) by which HO-1 inhibition might suppress pathogen growth is currently unclear. Heme triggers the production of reactive oxygen species (15), which are toxic to *M. tuberculosis* bacilli (23), and therefore, blockade of its degradation by HO-1 may result in higher production of these metabolites and consequently enhanced bacterial killing. Another possibility is that reduced heme degradation resulting from HO-1 inhibition may lead to diminished bacterial growth by limiting the availability of nutritionally required free iron (24).

Regardless of the exact mechanism involved, the data presented here reveal pharmacological inhibition of HO-1 activity as a candidate strategy for both direct and adjunctive therapy of tuberculosis. HO-1 inhibitors such as SnPPIX have already been used clinically in the treatment of jaundice in infants (25) and thus could potentially be repurposed for use in TB patients. However, further preclinical work is necessary to compare the efficacy of different HO-1 inhibitors, determine their optimal formulation (alone and in combination with conventional antibiotics), and confirm the safety of this new HDT strategy before considering human trials.

## SUPPLEMENTAL MATERIAL

Text S1  Supplemental methods and references. Download Text S1, PDF file, 0.1 MB

Table S1  SnPPIX does not significantly affect *M. tuberculosis* growth in liquid culture under normal or stress conditions. Table S1, PDF file, 0.04 MB

Table S2  Primers used for RT-PCR and real-time PCR. Table S2, PDF file, 0.03 MB

Figure S1  SnPPIX is not degraded by *M. tuberculosis* MhuD nor is its heme-degrading activity inhibited. (A) Degradation of heme and SnPPIX by MhuD was monitored by UV-vis spectroscopy every 5 min for 1 h for heme and for a period of 24 h for SnPPIX. Each color represents a different time point. (B) Heme degradation by MhuD in the presence of 2 μM [i.e., from the calculation of (absorbance of 5 μM MhuD-heme plus 2 μM SnPPIX) – (absorbance of 2 μM SnPPIX)] for each time point in order to correct for the absorbance in the presence of SnPPIX. (C) Heme degradation by recombinant human HO-1-G139A (hHO-1) in the absence or presence of SnPPIX (2 μM) was monitored every 5 min for 1 h. The data shown are either the absorbance (left) or the change in absorbance (Δabsorbance, as described in panel B, right panel). All experiments for panels A to C were performed in triplicate. (D) MhuD mRNA expression in lungs of C57BL/6 (WT) and TCR-α^−/−^ mice at 4 and 5 wpi. Results are expressed as mean femtograms per milliliter of cDNA per bacterium in each sample, ± the standard deviation (left) or as the ratio between average MhuD gene expression in WT and TCR-α^−/−^ mouse lung samples (right). Download Figure S1, PDF file, 1.7 MB

Figure S2  Delaying the adminstration of SnPPIX to a late phase following conventional antibiotic treatment fails to accelerate pulmonary bacterial clearance. (A) *M. tuberculosis*-infected C57BL/6 (WT) mice were left untreated or were treated with RHZ for the first 40 days starting at 4 wpi. The groups were then split into groups, and one half of the animals were treated with SnPPIX concurrently with the conventional antibiotics. Pulmonary CFU were quantified at the time points indicated. The dotted line represents the limit of detection for the assay. (B) Ratio of mean HO-1 mRNA expression in lungs of RHZ-treated versus untreated *M. tuberculosis*-infected mice at 3, 6, and 9 weeks post-treatment initiation (wpt). (C and D) Gamma interferon expression in CD4^+^ (C) and CD8^+^ (D) T lymphocytes in lung homogenates of untreated or RHZ-treated *M. tuberculosis*-infected mice at the indicated time points after the initiation of therapy. The cells were stimulated *in vitro* with phorbol myristate acetate and ionomycin for 5 h prior to staining. Download Figure S2, PDF file, 2.1 MB
