# Supplementary material for: Pharmacological Inhibition of Host Heme Oxygenase-1 Suppresses Mycobacterium tuberculosis Infection In Vivo by a Mechanism Dependent on T Lymphocytes
Source: mBio. 2016 Oct 25;7(5):e01675-16. doi: 10.1128/mBio.01675-16 (PMC5080384; doi:10.1128/mBio.01675-16)
Supplement: Text S1 — Supplemental methods and references. Download [file mbo005163040s1.pdf]

Supplemental Material and Methods

Assay for potential effects of SnPPIX on growth of *M. tuberculosis* in liquid culture

Two different assays were employed to determine whether SnPPIX is toxic for Mtb in culture. In the first, the minimum concentration of drug inhibiting 99% (MIC_{99}) of bacterial growth was determined using a previously described micro-dilution broth method (1), employing glycerol-alanine-salts (GAST) medium or GAST medium supplemented with either iron (GASTFe) or 10 μM hemin (GAST + hemin). Briefly, *M. tuberculosis* strain H37Rv was adapted to the Fe-sufficient and Fe-deficient media for 3 weeks with repeated sub-culturing once every week in fresh medium. The bacteria were then allowed to grow to an optical density (OD_{650}) of 0.2-0.3, diluted to a final OD_{650} of 0.0002 (1:1000 of parent culture) and distributed into a 96-well plate. SnPPIX was then added at 125μM, 62.5μM, 31.25μM, 15.63μM, 7.81μM, 3.9μM, 3.95μM, 0.98μM, 0.49μM, 0.24μM and 0.12μM to duplicate plates with each compound dilution further set-up in duplicate rows per plate. Cultures were incubated for 28 days at 37°C and the lowest concentration of drug that inhibited visible growth determined. Isoniazid was used as a positive control for anti-bacterial activity. Growth inhibition was also assessed in a second nitrosative stress assay wherein GFP-expressing *M. tuberculosis*/pMSP12 bacteria were adapted to 7H9 medium containing 250 μM butyrate (as the C-source) at pH 6.0 for 14 days. The inhibition assay was set up as described above but in the presence or absence of 100μM $NaNO_2$ rather than iron or hemin. On day 21 of culture, fluorescence was measured in an Envision multiplate reader (Perkin Elmer, Waltham, MA, USA) and the MIC_{95} values calculated as the drug concentration giving 95% inhibition of the fluorescent signal (fluorescence read in the presence of rifampicin was used as positive

control -100% inhibition, while flourescence in the presence of DMSO vehicle was used as negative control – 0% inhibition). Pyrazinamide is only active at low pH environment and therefore, inhibition of bacterial growth in its presence was used as a positive control for confirming maintenance of the low pH of the medium through the duration of the assay.

Recombinant MhuD and hHO-1 G139A protein expression and purification

Construction of the expression vector, pET22b-MhuD with a C-terminal His_6tag, and the expression and purification of *M. tuberculosis* MhuD have been previously described (2). In brief, MhuD was overexpressed in BL21-Gold (DE3) *Escherichia coli*. Cells were resuspended in 50 mM Tris/HCl pH 7.4, 350 mM NaCl and 10 mM imidazole and lysed by sonication. The cell supernatant was loaded onto a Ni^{2+}-charged HiTrap chelating column (5 mL) and washed with resuspension buffer. Fractions of eluted MhuD (between 50 and 100 mM imidazole) were collected and concentrated. Apo-MhuD was further separated on a S75 gel filtration column (GE Healthcare, Little Chalfont, UK) with 20 mM Tris, pH 8, and 10 mM NaCl before a final purification step with an ion exchange column (HiTrap Q HP, 5 mL) where homogeneous apo-MhuD eluted at 150 mM NaCl. Recombinant human heme oxygenase-1 variant G139A (hHO-1 G139A) clone was a gift from Dr. Thomas L. Poulos from the University of California, Irvine and was purified as previously described (3, 4).

MhuD-SnPPIX, MhuD-heme and hHO-1 G139A-heme / SnPPIX competition assays

For these assays, SnPPIX was dissolved in 300 µl of 0.1 M NaOH before dilution into 50 mM tris pH 7.4, 150 mM NaCl. The pH was readjusted back to 7.4 with 1 M HCl. Hemin was prepared by dissolving hemin chloride (Sigma-Aldrich) in 0.1 M NaOH

before the addition of 1 M Tris pH 7.4 and dilution into 50 mM Tris pH 7.4, 150 mM NaCl. To produce the MhuD-SnPPIX complex, SnPPIX was gradually added to 0.1 M Apo-MhuD in a 1:1 molar ratio. The mixture was incubated overnight at 4°C and exchanged into 50 mM Tris pH 7.4, 150 mM NaCl via a desalting column (HiTrap desalting, 5 mL). The protein concentration post-desalting was determined by Lowry assay. MhuD-heme was prepared as previously reported (5). Briefly, heme was gradually added to 0.1 M MhuD in a 1.2:1 molar ratio before overnight incubation at 4°C. Excess heme was removed using a desalting column (HiTrap desalting, 5 mL) and the eluted protein concentration was determined by Lowry assay. The human HO-1 variant G139A (hHO-1 G139A) was used as a positive control for heme degradation by the host enzyme as its reaction rate is attenuated by 58% (4), allowing for the observation of single turnover heme degradation within a similar time period as MhuD (2). The heme degradation reaction for hHO-G139A-heme was carried out in a similar manner as that for MhuD-heme. In all assays, sodium ascorbate was added as an electron donor to initiate the heme degradation process, as previously described (2). The reaction was monitored by UV/vis spectroscopy by collecting spectra between 300 – 700 nm at various time intervals and observing the decrease of the Soret peak over time to determine heme degradation. For the SnPPIX/heme competition assays, 2 μM SnPPIX was incubated with either 5 μM MhuD-heme or 5 μM apo-MhuD as well as 5 μM hHO-G139A-heme or 5 μM apo-hHO-1 G139A for 1 hr before addition of 10 mM sodium ascorbate to initiate the reaction. The difference spectra (ΔAbsorbance) were calculated by subtracting the reaction spectra without enzyme from those of the 5 μM enzyme-heme reaction to remove the absorbance interference of SnPPIX.

Real time PCR for bacterial MhuD

mRNA extraction and cDNA reverse transcription were performed as described in Material and Methods, except that cDNA was prepared using a specific primer for MhuD and a pre-amplification step of 10 cycles was performed prior to real time PCR reaction. A standard curve with known concentrations of bacterial MhuD cDNA was prepared and used to calculate the levels of MhuD expression in lung samples. The final cDNA concentration of each sample was then normalized to the number of CFU in the same samples. The primers sequences employed are shown in Table S2.

Isolation of cells from lung tissue and flow cytometric analysis of T cell populations

Single cell suspensions were obtained from lungs from Mtb-infected as previously described (6). Cells were stimulated with PMA (10 ng/ml) and ionomicin (1 μg/ml) in the presence of brefeldin A and monensin for 5 h prior to staining with specific fluorochrome-labelled antibodies and a fixable fluorescent viability dye (Molecular Probes/Thermo Fisher Scientific). The antibodies employed (obtained from either Affimetrix/ebioscience and BD biosciences (San Diego, CA, USA)) were directed against CD3 (clone 145-2C11), CD4 (clone GK1.5), CD8 (clone 53-6.7) and IFN-γ (clone XMG 1.2). All samples were acquired on LSRII flow cytometers (BD Biosciences) and analyzed utilizing FlowJo software (FlowJo LLC, Ashland, OR, USA).

Supplemental References

1. **Wong SY, Lee JS, Kwak HK, Via LE, Boshoff HI, Barry CE, 3rd.** 2011. Mutations in gidB confer low-level streptomycin resistance in *Mycobacterium tuberculosis.* Antimicrob Agents Chemother **55:**2515-2522.

2. **Chim N, Iniguez A, Nguyen TQ, Goulding CW.** 2010. Unusual Diheme conformation of the heme-degrading protein from *Mycobacterium tuberculosis*. J Mol Biol **395:**595-608.

3. **Wilks A, Ortiz de Montellano PR.** 1993. Rat liver heme oxygenase. High level expression of a truncated soluble form and nature of the meso-hydroxylating species. J Biol Chem **268:**22357-22362.

4. **Liu Y, Koenigs Lightning L, Huang H, Moenne-Loccoz P, Schuller DJ, Poulos TL, Loehr TM, Ortiz de Montellano PR.** 2000. Replacement of the distal glycine 139 transforms human heme oxygenase-1 into a peroxidase. J Biol Chem **275:**34501-34507.

5. **Tullius MV, Harmston CA, Owens CP, Chim N, Morse RP, McMath LM, Iniguez A, Kimmey JM, Sawaya MR, Whitelegge JP, Horwitz MA, Goulding CW.** 2011. Discovery and characterization of a unique mycobacterial heme acquisition system. Proc Natl Acad Sci U S A **108:**5051-5056.

6. **Mayer-Barber KD, Andrade BB, Oland SD, Amaral EP, Barber DL, Gonzales J, Derrick SC, Shi R, Kumar NP, Wei W, Yuan X, Zhang G, Cai Y, Babu S, Catalfamo M, Salazar AM, Via LE, Barry CE, 3rd, Sher A.** 2014. Host-directed therapy of tuberculosis based on interleukin-1 and type I interferon crosstalk. Nature **511:**99-103.
