# Supplementary material for: Pharmacological Inhibition of Host Heme Oxygenase-1 Suppresses Mycobacterium tuberculosis Infection In Vivo by a Mechanism Dependent on T Lymphocytes
Source: mBio. 2016 Oct 25;7(5):e01675-16. doi: 10.1128/mBio.01675-16 (PMC5080384; doi:10.1128/mBio.01675-16)
Supplement: Table S1 — SnPPIX does not significantly affect M. tuberculosis growth in liquid culture under normal or stress conditions. [file mbo005163040st1.pdf]

Table S1: SnPPIX does not significantly affect *M. tuberculosis* growth in liquid culture under normal or stress conditions.

Experiment 1 – day 28 MIC_{99}		
	SnPPIX (μM)	Isoniazid (μM)
GASTFe	>125	0.2
GAST	125	0.1
GAST + 10μM Hemin	>125	0.1

Experiment 2 – day 21 MIC_{95}		
	SnPPIX (μM)	Rifampicin (μM)
7H9-butyrate	>125	0.04
7H9-butyrate + 100μM $NaNO_2^-$	>125	0.04
