# Supplementary material for: Pharmacological Inhibition of Host Heme Oxygenase-1 Suppresses Mycobacterium tuberculosis Infection In Vivo by a Mechanism Dependent on T Lymphocytes
Source: mBio. 2016 Oct 25;7(5):e01675-16. doi: 10.1128/mBio.01675-16 (PMC5080384; doi:10.1128/mBio.01675-16)
Supplement: Table S2 — Primers used for RT-PCR and real-time PCR. [file mbo005163040st2.pdf]

Table S2: Primers used for RT-PCR and real time PCR

	Forward primer (5'- 3')	Reverse primer (5'- 3')
Murine Actb	AGC TGC GTT TTA CAC CCT TT	AAG CCA TGC CAA TGT TGT CT
Murine Hmox1	GCC ACC AAG GAG GTA CAC AT	GCT TGT TGC GCT CTA TCT CC
Mtb Rv3592 (MhuD) - cDNA		TTA TGC AGT CTT GCC GGT CC
Mtb Rv3592 (MhuD) - real time PCR	AAC GCT ACT TCG TGG TGA CA	CGT CAA GCA CGA CCT CGA AT
