# Supplementary material for: Pharmacological Inhibition of Host Heme Oxygenase-1 Suppresses Mycobacterium tuberculosis Infection In Vivo by a Mechanism Dependent on T Lymphocytes
Source: mBio. 2016 Oct 25;7(5):e01675-16. doi: 10.1128/mBio.01675-16 (PMC5080384; doi:10.1128/mBio.01675-16)
Supplement: Figure S1 — SnPPIX is not degraded by M. tuberculosis MhuD nor is its heme-degrading activity inhibited. (A) Degradation of heme and SnPPIX by MhuD was monitored by UV-vis spectroscopy every 5 min for 1 h for heme and for a period of 24 h for SnPPIX. Each color represents a different time point. (B) Heme degradation by MhuD in the presence of 2 μM [i.e., from the calculation of (absorbance of 5 μM MhuD-heme plus 2 μM SnPPIX) – (absorbance of 2 μM SnPPIX)] for each time point in order to correct for the absorbance in the presence of SnPPIX. (C) Heme degradation by recombinant human HO-1-G139A (hHO-1) in the absence or presence of SnPPIX (2 μM) was monitored every 5 min for 1 h. The data shown are either the absorbance (left) or the change in absorbance (Δabsorbance, as described in panel B, right panel). All experiments for panels A to C were performed in triplicate. (D) MhuD mRNA expression in lungs of C57BL/6 (WT) and TCR-α−/− mice at 4 and 5 wpi. Results are expressed as mean femtograms per milliliter of cDNA per bacterium in each sample, ± the standard deviation (left) or as the ratio between average MhuD gene expression in WT and TCR-α−/− mouse lung samples (right). Download [file mbo005163040sf1.pdf]

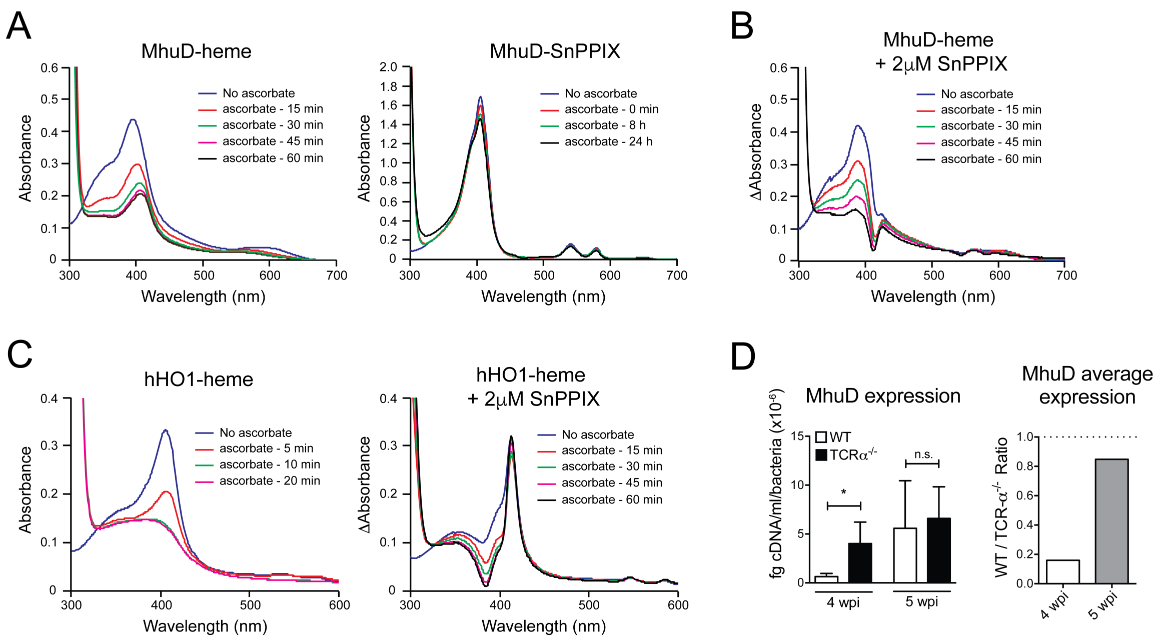

Figure S1. SnPPIX is not degraded by Mtb MhuD nor inhibits its heme degrading activity. (A) Degradation of heme and SnPPIX by MhuD was monitored by UV/vis spectroscopy every 5 min for 1h for heme and for a period of 24h for SnPPIX. Each color represents a different time point. (B) Heme degradation by MhuD in the presence of 2 μM SnPPIX was monitored every 5 min for 1h. Data shown are expressed as the ΔAbsorbances ([absorbance of 5 μM MhuD-heme + 2 μM SnPPIX] – [absorbance of 2 μM SnPPIX]) for each time point in order to correct for the absorbance in the presence of SnPPIX. (C) Heme degradation by recombinant human HO-1-G139A (hHO-1) in the absence or presence of SnPPIX (2 μM) was monitored every 5 min for 1h. The data are shown as either absorbance (left panel) or change in absorbance (ΔAbsorbance, as described in panel B, right panel). All experiments for panels A, B and C were performed in triplicate. (D) MhuD mRNA expression in lungs of C57BL/6 (WT) and TCR-$\alpha^{-/-}$ mice at 4 and 5 wpi. Results are expressed as mean femtograms/ml of cDNA per bacteria in each sample ± standard deviation (left panel) and as the ratio between the average MhuD gene expression in WT and TCR-$\alpha^{-/-}$ mouse lung samples (right panel).
