# Supplementary material for: Pharmacological Inhibition of Host Heme Oxygenase-1 Suppresses Mycobacterium tuberculosis Infection In Vivo by a Mechanism Dependent on T Lymphocytes
Source: mBio. 2016 Oct 25;7(5):e01675-16. doi: 10.1128/mBio.01675-16 (PMC5080384; doi:10.1128/mBio.01675-16)
Supplement: Figure S2 — Delaying the adminstration of SnPPIX to a late phase following conventional antibiotic treatment fails to accelerate pulmonary bacterial clearance. (A) M. tuberculosis-infected C57BL/6 (WT) mice were left untreated or were treated with RHZ for the first 40 days starting at 4 wpi. The groups were then split into groups, and one half of the animals were treated with SnPPIX concurrently with the conventional antibiotics. Pulmonary CFU were quantified at the time points indicated. The dotted line represents the limit of detection for the assay. (B) Ratio of mean HO-1 mRNA expression in lungs of RHZ-treated versus untreated M. tuberculosis-infected mice at 3, 6, and 9 weeks post-treatment initiation (wpt). (C and D) Gamma interferon expression in CD4+ (C) and CD8+ (D) T lymphocytes in lung homogenates of untreated or RHZ-treated M. tuberculosis-infected mice at the indicated time points after the initiation of therapy. The cells were stimulated in vitro with phorbol myristate acetate and ionomycin for 5 h prior to staining. Download [file mbo005163040sf2.pdf]

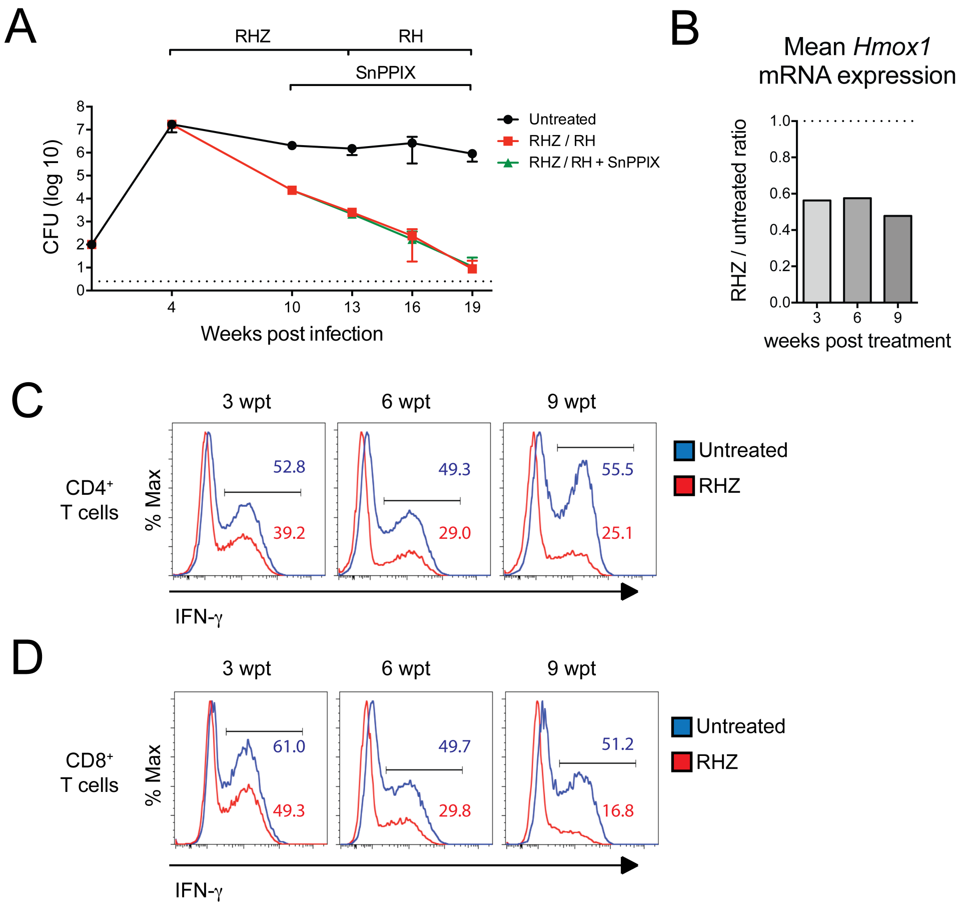

Figure S2. Delaying the administration of SnPPIX to a late phase following conventional antibiotic treatment fails to accelerate pulmonary bacterial clearance. (A) Mtb-infected C57BL/6 (WT) mice were left untreated or were treated with RHZ for the first 40 days starting at 4 wpi. The groups were then split and one half of the animals treated with SnPPIX concurrently with the conventional antibiotics. Pulmonary CFU were quantitated at the time points indicated. The dotted line represents the limit of detection for the assay. (B) Ratio of the mean HO-1 mRNA expression in lungs of RHZ treated vs untreated Mtb-infected mice at 3, 6 and 9 wks post-treatment (wpt) initiation. (C and D) IFN-γ expression in $CD4^+$ (C) and $CD8^+$ T (D) lymphocytes in lung homogenates of untreated or RHZ treated Mtb-infected mice at indicated time points after the initiation of therapy. The cells were stimulated in vitro with PMA and ionomycin for 5h prior to staining.
